# Roles of participation in social activities in the association between adverse childhood experiences and health among older Japanese adults

**DOI:** 10.1016/j.ssmph.2021.101000

**Published:** 2021-12-14

**Authors:** Marisa Nishio, Michael Green, Naoki Kondo

**Affiliations:** a School of Social & Political Sciences, University of Glasgow, Glasgow G12 8QQ, Scotland, UK; b MRC/CSO Social and Public Health Sciences Unit, University of Glasgow, Berkeley Square, 99 Berkeley Street, Glasgow, G3 7HR, Scotland, UK; c Department of Social Epidemiology, Graduate School of Medicine and School of Public Health, Kyoto University, Floor 2, Science Frontier Laboratory, Yoshida-konoe-cho, Sakyo-ku, Kyoto-shi, Kyoto, 606-8315, Japan

**Keywords:** Social participation, Adverse childhood experience, Older adults, Controlled direct effect, Depression, Subjective health

## Abstract

Adverse childhood experiences (ACEs) have shown strong associations with later-life health such as depression and subjective health. Social participation is also associated with later-life health but it is unclear to what extent this could contribute to alleviating harmful impacts of ACEs, nor is it clear whether ACEs are themselves associated with later-life social participation. Thus, this study aims to understand: (1) the influence of ACEs on social participation in later life and (2) whether social participation can alleviate the harmful influences of ACEs on depression and subjective health among Japanese older adults. Data were from 5,671 Japanese older adults (aged 65+) in surveys in 2013 and 2016 as part of the Japan Gerontological Evaluation Study (JAGES). Logistic regression analyses were conducted to estimate the relations between ACEs and later-life social participation, adjusting for potential confounders and mediators. Inverse probability weighting was used to estimate average effects of ACEs on later-depression and subjective health, adjusting for potential confounders, and these were compared against controlled direct effect (CDE) estimates from marginal structural models based on all respondents experiencing weekly social participation. We found that ACEs were associated with reduced later-life social participation (OR for >1 ACEs = 0.88, 95% CI = 0.79, 0.99). The estimated effect of ACEs on depression ( adjusted total effect estimates: OR = 1.23, 95% CI = 1.05, 1.45) was marginally alleviated in estimates assuming weekly social participation for everyone (CDE = 1.18, 95% CI = 0.98, 1.43). A similar tendency was seen for poor subjective heath. Negative impacts of ACEs on depression may be marginally mitigated through social participation, but mitigating effects were moderate. Further investigation on other potential later-life mitigating factors is needed.

## Highlights

• Adverse childhood experiences (ACEs) are related to lower later-life sociability.
• Such inequality in sociability may result in wider health disparities.
• Weekly social participation may moderately mitigate impacts of ACEs on depression.
• Further study is needed on other potential means of mitigating the impact of ACEs.

## 1 Introduction

Population ageing is expanding worldwide. In 2019, 703 million people were aged 65 years or over, which was 10% of the global population (UN Population Division, 2019). Countries will continue to face increasingly older populations until 2050 when they are expected to reach 1.5 billion or more. Japan is the leading country worldwide in the speed of population ageing and is projected to maintain the world's highest ratio of people aged 65 years or over to those of working age until at least 2050 (projected at 81 per 100) (UN Population Division, 2019). The maintenance of physical, mental and social capabilities of older people has become a significant target for public health interventions (World Health Organization, 2002), which are especially relevant to Japan and its ageing population. This study explores social participation as a means of mitigating impacts of adverse early life experiences on later-life health and depression.

In life course epidemiology, early life experiences are understood to be important determinants of later-life health (Cheong et al., 2017; Cristóbal-Narváez et al., 2016; Hughes et al., 2016, 2017; Nurius et al., 2012; Shawi et al., 2019; Westreich & Greenland, 2013), and there is a growing recognition of the influence of Adverse Childhood Experiences (ACEs). ACEs are ‘potentially traumatic events or chronic stressors that occur before the age of 18 and are uncontrollable to the child’(Felitti et al., 1998). They can include both direct harm (e.g. violence, financial difficulties and neglect) and indirect harm (e.g. mental illness and parental conflict) (Edwards, 2019; Hughes et al., 2017). Older people who have had ACEs are more likely to experience depression and have lower subjective health (Cheong et al., 2017; Tani, Fujiwara, et al., 2016; Tani, Kondo, et al., 2016), and with populations continuing to age globally, the development of public/global health interventions that can mitigate such effects in old age is especially important (World Health Organization, 2015). Older people with a history of childhood maltreatment tend to have additional medical and care costs compared with those who have not had ACEs (Bellis et al., 2019; Isumi et al., 2020), so any mitigating interventions could have important impacts in reducing healthcare costs. There is evidence that social participation in neighbourhood activities can enhance the health of older people (Levasseur et al., 2010; Saito et al., 2019; World Health Organization, 2002). For instance, many studies have found that social participation was associated with reduced risk of depression (Takagi et al., 2013), poor subjective health (Leone & Hessel, 2015), cognitive decline (Zunzunegui et al., 2003), mortality (Glass et al., 1999; Hsu, 2007) and impaired physical function (Kanamori et al., 2014). This might be because social participation can create a network of relationships and intimate ties, allowing access to actual, expressive and crisis support, facilitating mental well-being (Lin et al., 1999).

In the absence of intervention, however, ACEs could actually influence the extent of later-life social participation. This is because prior empirical studies show that a favourable family environment, such as warm, sensitive parenting in childhood, predicts adaptive personality traits and temperament in later-life (Eisenberg et al., 2015; Josefsson et al., 2013; Kazuhisa et al., 2000). Moreover, childhood experience of ACEs was significantly associated with later behavioral problems from childhood to adolescence (Choi et al., 2019). Thus, if ACEs hinder the development of sociability, people with adverse experiences could find it more difficult to participate in social activities, and this could contribute to or widen the health disadvantage of those with ACEs. Alternatively, social participation may be a coping strategy used by those who have experienced ACEs to foster resilience and recovery (Bonanno et al., 2011; Southwick et al., 2014), so social participation in later life could be more frequent for those who have experienced ACEs.

Nevertheless, it is unclear how strongly ACEs are related to sociability in later-life and unclear to what extent social participation can mitigate the impact of ACEs. Thus, this study aims to understand: (1) the influence of ACEs on social participation in later life and (2) whether social participation can alleviate the harmful influences of ACEs on depression and subjective health among Japanese older adults (both known as later-life health outcomes associated with ACEs (Hughes et al., 2017)).

## 2 Methods

### 2.1 Data

**Fig. 1:**
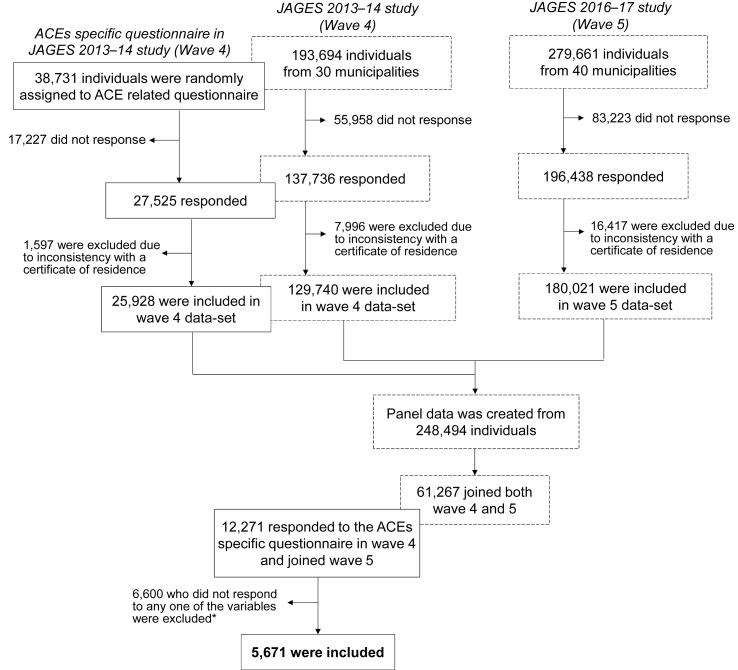
Flowchart showing the structure of JAGES 13–16 panel dataset and selection of participants.

Data were from the Japan Gerontological Evaluation Study (JAGES). The JAGES Project is an ongoing nationwide survey to understand the physical and mental health, socioeconomic status and health behaviour of community-dwelling older adults in Japan (Kondo et al., 2018). Ethics approval for the JAGES study was obtained from the ethical committee at the National Centre for Geriatrics and Gerontology (approval no. 992) and Chiba University (approval no. 2493). This study used data from waves four (in 2013) and five (in 2016) because these included more municipalities with better response rates than previous waves. In wave four, random sampling was conducted by local government officials in each of 4 large municipalities, while all eligible residents in 14 small municipalities and one region received the questionnaire. The remaining 13 municipalities mailed questionnaires to their pre-wave 4 participants and newly eligible participants (newly aged 65 and over). A total of 137,736 participants gave valid data (overall response rate: 71.1%). JAGES consists of a core questionnaire for all respondents with more specific and sensitive content, such as on ACEs and disability, in additional modules. The additional modules were randomly allocated within each municipality as follows: first, extracting older people who were born before 1 April 1948 and who live independently (i.e. those who do not use care services) as the eligible population; second, making a list of eligible population sorted by postcode and age in ascending order; third, the additional modules A to E were allocated within each municipality by consecutive assignment to every fifth respondent from a list of all those eligible, sorted by postcode and age. From participants assigned to the ACEs module in wave four, those who also participated at wave five were extracted (n = 12,271). Data from 5,671 people was used for analysis, after excluding 6,600 who did not complete all questions of interests (Fig. 1). Response rates are shown in supplementary appendix 1 (Figure A.1).

### 2.2 Measurement

#### 2.2.1 Depressive symptoms

Depressive symptoms were reported in 2016 using a short version of the Geriatric Depression Scale (GDS-15) (Almeida & Almeida, 1999). In accordance with previous studies (Saito et al., 2014; Takagi et al., 2013), a score of 0–4 was considered ‘no depression’, while those with a score of 5 or more were considered as possibly ‘depressed’ (see supplementary appendix 4 for full texts of the questions).

#### 2.2.2 Subjective health

In 2016, participants were asked ‘*How is your current health status?’* and had to choose from ‘*Excellent’*, ‘*Good*’, ‘*Fair*’ or ‘*Poor*’. The variables were dichotomised into ‘good’ (excellent or good) and ‘not good’ (fair or poor).

#### 2.2.3 ACEs

ACEs were reported retrospectively in 2013–14 (wave 4). Participants were asked ‘*Did you experience any of the following before the age of 18?’* and had to answer ‘*Yes’* or ‘*No*’ to each item from a list of potential ACEs as follows (translated from Japanese): (1) *Loss of parent(s)*; (2) *Parents' divorced*; (3) *Parent(s) suffered a mental illness*; (4) *Father was violent with mother*; (5) *Was hit hard by parent(s) causing an injury*; (6) *Felt loved by parents* (counted ‘No’ rather than ‘Yes’ for this item); (7) *Was told hurtful things or was insulted by parent(s)*; and (8) *Had financial trouble*. The variable was dichotomised in accordance with whether any ACEs were reported.

#### 2.2.4 Later-life sociability

Respondents were asked ‘*The following questions are about clubs, groups, and jobs you are currently engaged in. How often do you attend activities for the following groups*.’ The types of activities were: ‘*volunteer groups*’, ‘*sports groups*’, ‘*hobby groups*’, ‘*senior citizen clubs*’ and ‘*neighbourhood associations*’. A binary variable was created to indicate participation at least once a week in at least one group or organisation.

#### 2.2.5 Covariates

Sex, age and childhood SES were identified as potential confounders of ACEs and later-life social participation. In 2016–2017 (wave 5), respondents were asked ‘*What do you think of your living condition at the age of 15 in light of the social average around you?’* with five choices: ‘*Upper’*, ‘*Upper middle*’, ‘*Middle*’, ‘*Lower middle’* and ‘*Lower*’. This was taken as an indicator of childhood socioeconomic status (SES). The questions on ACEs enquired about experiences before 18 years of age, and this overlap in the timing of measures means it is unclear whether childhood SES preceded or was preceded by any ACEs experienced.

**Fig. 2:**
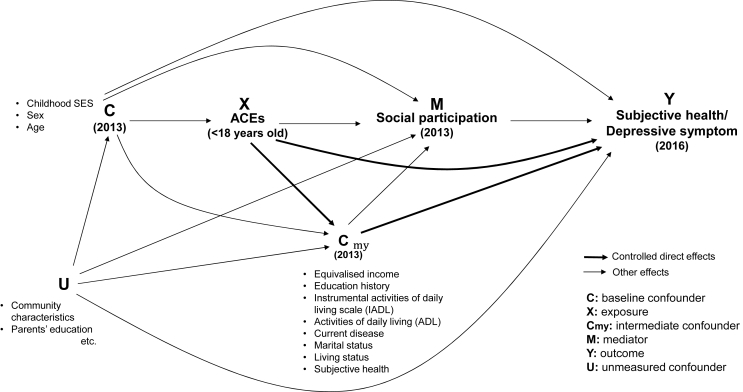
DAG

Several potential confounders of the association between later-life social participation (in wave four) and health (in wave five) were also controlled: SES i.e. equivalised income (Japanese yen: annual household income reported in categories was equivalised for household size and then coded in tertiles) and education history (years: <9, 10–12, >13, other); instrumental activities of daily living scale (IADL: <5 (low disability) vs ≧ 5 (high disability)); activities of daily living (ADL: no need of nursing care vs needs nursing care/receives nursing care); current disease (no disease vs currently receiving treatment or experiencing at least one of a list of common diseases); marital status (married vs widowed/divorced/never married/other); living status (living alone vs living with others); subjective health (very good/good vs not good/bad); depressive symptoms (GDS 15: <5 (no depression) vs ≧ 5 (depression)). These confounders may also be potential mediators between ACEs and later-life social participation (Fig. 2). We repeated analyses stratified by age (<68 vs 68+) to see if results differed depending on whether respondents were born after world war II, but findings were consistent (data not shown).

### 2.3 Statistical analysis

The associations between ACEs and social participation were estimated by logistic regression and results are presented as odds ratios (OR) with 95% confidence intervals (CI). Covariate adjustment was performed in four, cumulative stages: (1) sex and age; (2) childhood SES; (3) socio-demographic backgrounds and adult SES; and (4) physical and mental health. Although the confounder adjusted Model 2 might be the best estimate for the effect of ACEs on adult sociability, Models 3 and 4 were conducted to estimate direct effects of ACEs, not via these mediators. This is useful for comparing with other studies that have adjusted for potential mediators (Kelly-Irving & Delpierre, 2019). However, such adjustment may induce collider bias (Greenland, 2003) on the ACEs-sociability relationship if there are unmeasured confounders of the relationship between these mediators and later-life sociability.

In order to assess the extent to which a relationship between ACEs and later-life health can be modified by later-life social participation, counterfactual mediation analysis was then conducted (see Fig. 2 for DAG).

We compare three estimates: (1) an unadjusted association between ACEs and the outcome; (2) a confounder adjusted total effect estimate of ACEs on the outcome; and (3) a controlled direct effect (CDE) estimate where social participation is fixed to ‘weekly for everyone’ (along with adjustment for confounders). More traditional mediation methods can be subject to well-known biases related to exposure-mediator interaction and exposure-induced mediator-outcome confounding (Richiardi et al., 2013; VanderWeele, 2009). Our CDE estimates account for these issues and focus on what we are most interested in (Naimi et al., 2014; VanderWeele, 2009), namely the effect of ACEs after controlling social participation to the same level for everyone.

In second and third models, the inverse probability-weighted (IPW) method was adopted to remove imbalance that is not caused by exposure (Hernán & Robins, 2020). Predicted probabilities were extracted from logistic regression models predicting the exposure of interest (ACEs) and the mediator (social participation) and analysis weights were calculated and employed to obtain the average effect and CDE estimates (see supplementary appendix 2 for details). The CDEs of ACEs on depressive symptoms and subjective health were obtained by inverse-probability weighted logistic regression models including ACEs, social participation and their interaction terms. Each CDE was interpreted as the remaining ‘direct’ effect of the ACEs (i.e. not through social participation) under a hypothetical intervention setting social participation to a particular level (i.e. social participating once a week in at least one group or organisation) for all participants. All analyses were performed using Stata version 14.2 (StataCorp, College Station, TX, USA).

## 3 Results

### 3.1 Descriptive analysis

Descriptive analysis (Table 1) showed that the mean numbers of ACEs were 0.978 ([SD = 0.97]) for males and 0.782 ([SD = 0.91]) for females. Financial trouble was the most frequent ACE in both males and females (50.7% and 38.52%, respectively). Weekly participation in social activities was more prevalent among females: (male vs female: 31.05% vs 43.16%). The proportion of participants with depression and poor subjective health were about the same for men and women (19.57% male and 20.24% female for the former; 15.54% male and 12.21% female for the latter).

**Table 1:** Descriptive analysis.

	Overall (n = 5,671)		Male (n = 2,953)		Female (n = 2,718)	
	Mean	SD	Mean	SD	Mean	SD
**Age in 2016**	75.08	5.28	75.08	5.33	75.07	5.23
**Number of Adverse Childhood Experiences (ACEs) in total**	0.88	0.94	0.98	0.97	0.78	0.91
	*N*	*%*	*N*	*%*	*N*	*%*
**ACEs in each**						
*Loss of a parent*						
Experienced	1,216	21.44	632	21.40	584	21.49
Not experienced	4,455	78.56	2,321	78.60	2,134	78.51
*Parents' divorce*						
Experienced	99	1.75	56	1.90	43	1.58
Not experienced	5,572	98.25	2,897	98.10	2,675	98.42
*Mother or father suffered a mental illness*						
Experienced	36	0.63	22	0.75	14	0.52
Not experienced	5,635	99.37	2,931	99.25	2,704	99.48
*Your father was violent with your mother*						
Experienced	213	3.76	121	4.10	92	3.38
Not experienced	5,458	96.24	2,832	95.90	2,626	96.62
*Was hit hard by your mother/father causing an injury*						
Experienced	51	0.90	40	1.35	11	0.40
Not experienced	5,620	99.10	2,913	98.65	2,707	99.60
*Felt not loved by your parents*						
Experienced	585	10.32	369	12.50	216	7.95
Not experienced	5,086	89.68	2,584	87.50	2,502	92.05
*Was told hurtful things or was insulted by your mother/father*						
Experienced	269	4.74	151	5.11	118	4.34
Not experienced	5,402	95.26	2,802	94.89	2,600	95.66
*Financial trouble*						
Experienced	2,545	44.88	1,498	50.73	1,047	38.52
Not experienced	3,126	55.12	1,455	49.27	1,671	61.48
**Childhood SES**						
Upper	793	13.98	316	10.70	477	17.55
Middle	2,612	46.06	1,280	43.35	1,332	49.01
Lower	2,266	39.96	1,357	45.95	909	33.44
**Frequency of social participation in 2013**						
≧1 per week	2,090	36.85	917	31.05	1,173	43.16
<1 per week	3,581	63.15	2,036	68.95	1,545	56.84
**Depressive symptoms in 2016**						
Depression	1,128	19.89	578	19.57	550	20.24
No depression	4,543	80.11	2,375	80.43	2,168	79.76
**Subjective health in 2016**						
Bad	81	1.43	50	1.69	31	1.14
Not good	631	11.13	363	12.29	268	9.86
Good	4,146	73.11	2,127	72.03	2,019	74.28
Very good	813	14.34	413	13.99	400	14.72
**Equivalised income in 2013 (average: 232,3805 JPY)**						
Higher than average	2,748	48.46	1,521	51.51	1,227	45.14
Lower than average	2,923	51.54	1,432	48.49	1,491	54.86
**Education history in 2013**						
<9 years	1,776	31.32	817	27.67	959	35.28
10–12 years	2,376	41.90	1,145	38.77	1,231	45.29
>13 years	1,491	26.29	975	33.02	516	18.98
Others	28	0.49	16	0.54	12	0.44
**Activities of daily living (ADL) in 2013**						
Low ADL	808	14.25	611	20.69	197	7.25
High ADL	4,863	85.75	2,342	79.31	2,521	92.75
**Instrumental activities of daily living scale (IADL) in 2013**						
Low IADL	48	0.85	26	0.88	22	0.81
High IADL	5,623	99.15	2,927	99.12	2,696	99.19
**Current disease in 2013**						
Having no disease	1,121	19.77	575	19.47	546	20.09
Having 1 or more	4,550	80.23	2,378	80.53	2,172	79.91
**Marital status in 2013**						
Married or was married	1,193	21.04	279	9.45	914	33.63
Never married or other	4,478	78.96	2,674	90.55	1,804	66.37
**Living status in 2013**						
Living alone	621	10.95	187	6.33	434	15.97
Living with others	5,050	89.05	2,766	93.67	2,284	84.03

The basic sex- and age-adjusted model showed that experience of one or more ACEs was associated with an odds ratio (OR) for participating in community activities more than once a week of 0.88 (95% confidence interval [CI] = [0.79, 0.99]), compared with those having not experienced any ACEs (Model 1, Table 2). However, these associations were not statistically significant after adjusting for childhood SES (Model 2, Table 2) and were further attenuated with adjustment for other potential later-life mediators.

**Table 2:** Multivariate logistic regression models for the association of more than one ACE and later-life social participation.

	Model 1		Model 2		Model 3		Model 4	
	OR	95% CI	OR	95% CI	OR	95% CI	OR	95% CI
No ACE	Reference		Reference		Reference		Reference	
>1ACEs	0.88	[0.79, 0.99]	0.96	[0.85,1.09]	1.01	[0.89,1.14]	1.07	[0.94,1.21]

Notes: OR denotes odds ratio, CI indicates confidence interval.

Model 1: adjusted for sex and age.

Model 2: adjusted for potential confounder, i.e. childhood SES.

Model 3: adjusted for potential confounder and mediators in relation to socio-demographic backgrounds and adulthood SES, i.e. marital status, household status, education history, current income and occupation.

Model 4: adjusted for childhood SES, socio-demographic backgrounds and adulthood SES and potential mediators in relation to physical and mental health, i.e. subjective health, depressive symptoms, current disease, ADL and IADL.

**Fig. 3:**
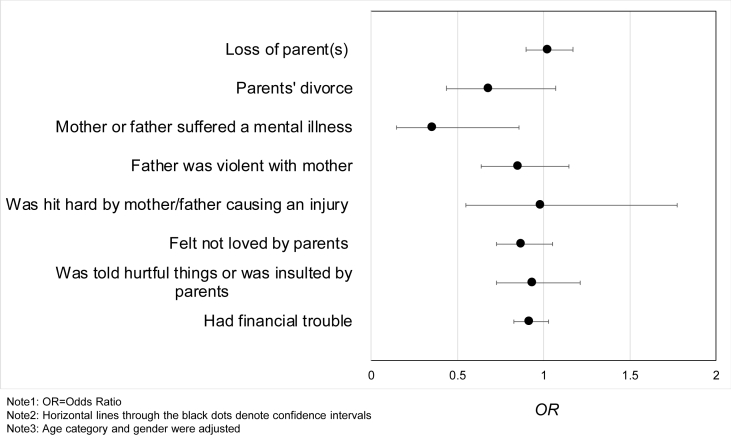
Forest plot of the association between each ACE and frequent social participation. Note1: OR=Odds Ratio. Note2: Horizontal lines through the black dots denote confidence intervals. Note3: Age category and gender were adjusted.

In addition, the association between later-life social participation and ACEs varied by the types of ACEs (Fig. 3).

Participants who had a parent with a mental illness in childhood had reduced odds of weekly social participation in later life (OR = 0.35, 95% CI [0.15, 0.86]) (see appendix 3 for detail). None of the other types of ACE showed a clear, independent relationship, though many had ORs lower than 1.

Table 3 shows the unadjusted estimates, adjusted total effect estimates and CDE estimates for the effect of one or more ACEs on depressive symptoms and subjective health in each. CDE estimates were adjusted for confounders and fixed social participation to weekly for everyone.

**Table 3:** Estimates of effects of ACEs on depressive symptoms and health (social participation at least once a week).

	Unadjusted association		Adjusted total effect estimate		CDE	
*Outcomes*	*OR*	*95% CI*	*OR*	*95% CI*	*OR*	*95% CI*
Depressive symptoms	1.45	[1.26,1.66]	1.23	[1.05,1.45]	1.18	[0.98,1.43]
Subjective health	1.46	[1.25,1.71]	1.20	[0.99,1.45]	1.18	[0.95,1.46]

Notes: OR denotes odds ratio, CI indicates confidence interval.

The unadjusted associations indicate that having any ACE was associated with a greater likelihood of having depression in later life with statistical significance (OR = 1.45, 95% CI = 1.26,1.66). After adjusting for confounders, the total effect estimate was weaker but still present (OR = 1.23, 95% CI [1.05, 1.45]). CDE estimates of ACEs on depression (setting all participants to weekly social participation) were more attenuated (CDE: OR = 1.18, 95% CI [0.98, 1.43]). A similar tendency was seen in all estimates, including CDEs, on subjective health.

## 4 Discussion

We present empirical evidence from a large cohort study of Japanese older adults regarding the correlation between ACEs and later-life sociability and health. ACEs were associated with a lower frequency of social participation in later life, and that one particular ACE — mother or father suffered a mental illness — was most clearly associated with a reduced probability of later-life social participation. Further, it was estimated that weekly social participation in later life could only marginally alleviate the negative influence of ACEs on both depression and subjective health.

Our findings are consistent with others who have found that childhood adversities were associated with reduced later-life social engagement, as indicated by part-time employment and retirement in those age 55 years old (Fahy et al., 2017). Our study demonstrates that ACEs also affect participation in community social activities, including hobbies and sports. In Japan, health promotion policies encourage older people participating in community social activities (Minister of Health, 2012), which may increase the gap in social participation between those with and without ACE.

However, in our study, introducing a childhood SES adjustment attenuated the estimated association between ACEs and social participation. This might be a case of over-adjustment. The available measure of childhood SES concerned individuals' socioeconomic status (or household conditions) when they were 15 years old, while respondents were asked to report ACEs from infancy up to age 18. Therefore, the overlapping measurement period might mean childhood SES at 15 was caused by ACEs earlier in life, which would position it as a mediator rather than a confounder of the association between ACEs and later-life social participation. Single parents, for example, tend to have lower incomes (Maldonado & Nieuwenhuis, 2015). Assuming that parental divorce, as an ACE, induces low childhood SES and less frequent social participation in later life, it can be considered that childhood SES mediates the relationship. An alternative explanation of this attenuation is that childhood socioeconomic disadvantage is a determinant of ACEs, and then influences later-life social participation (i.e. classic confounding). A systematic review conducted by Walsh et al. suggested that lower childhood socioeconomic position was often associated with a greater risk of having had ACEs (Walsh et al., 2019), which makes this confounding explanation plausible.

Decreased likelihood of participating in social activity among those who had a mother or father with mental illness is also consistent with the results of prior studies on the unfavourable impact of parents with mental illness on child development (Cooklin, 2009; Stein et al., 2014). Poor parental mental health affects children in many ways. For example, the responsibility of caring for a parent with mental illness and/or sibling becomes a burden on the child, which can prevent he/she from attending age-appropriate activities and school (Maybery et al., 2005). In fact, Mensah at al. suggest that children whose parents have high levels of psychological distress show lower achievements in communication, language and literacy; disrupted development of personal, social and emotional skills; and lower mathematical skills' attainment (Mensah & Kiernan, 2009). Although parental age, qualifications and socioeconomic status mediate the relations between parental psychological distress and children's educational achievements, independent effects of maternal mental health are observed (Mensah & Kiernan, 2009). As such, poor parental mental health might limit educational and/or social opportunities in childhood, with follow-on effects leading to impacts on later-life sociability.

Compared to the confounder adjusted models, CDE estimates that adjusted for confounders and fixed social participation to ‘weekly for everyone’ marginally attenuated the frequency of having depression and had relatively little impact on poor subjective health, suggesting that interventions ensuring everyone gets weekly socialization would have limited scope for mitigating the impacts of ACEs. This is somewhat consistent with reports of the mediating effects of perceived social support in the relationship between ACEs and depression (Cheong et al., 2017), but emphasizes that only marginal gains are likely to be achieved by intervening to ensure weekly socialization. To conclude that later-life social participation does not contribute to alleviating the negative effects of ACEs, further studies are necessary to evidence that there is no effect at all in any frequency of social participation. Alternatively, it may be more important to prioritise identifying more substantial mitigating effects via other public health interventions, such as providing additional social welfare to people who have experienced traumatic childhood events.

In this study, a large sample of population-based panel data was used, which enabled the use of conditional causal mediation analysis by adopting marginal structural modelling with inverse probability-weighted methods. This helped overcome some potential biases present in traditional mediation analyses that are associated with adjustment for mediator-outcome confounders that are caused by exposure and exposure-mediator interaction (Naimi et al., 2014; Richiardi et al., 2013; VanderWeele, 2009).

This study has some limitations. Given the nature of self-reported retrospective surveys, the data concerning ACEs and childhood SES may be susceptible to recall bias (Kelly-Irving & Delpierre, 2019). If, for example, more adverse experiences are more easily recalled, then the true association between childhood SES and ACEs may be accentuated in the data, and the impact of controlling for childhood SES may be over-estimated. Furthermore, reverse causation between ACEs and later-life social participation and/or later-life SES could not be ruled out. Even though the questionnaire asked participants about their experiences when they were younger than 18, it is possible, for example, that those who participate in social activities are more willing to talk about and recall their adverse childhood experiences. It is also possible that people who are economically deprived (i.e., who have low SES) may be more likely to remember their adverse experiences. Finally, the association between ACEs and later-life social participation and health could also reflect other omitted variables, such as individual characteristics, the inability to communicate with others, temperament, personality or later-life traumatic events, which are not measured in the JAGES study.

## 5 Conclusion

Having any one of the ACEs was associated with reduced social participation in later-life, but this association was not independent of childhood SES or other later-life characteristics. Such inequality in social participation may result in a wider health imbalance between people with and without ACEs. The negative impact of ACEs on later-life health may be marginally mitigated through promoting social participation, but estimated mitigating effects were limited. Prevention and early interventions for ACEs are vital, although understanding effective later-life interventions are important to help reduce disparities among those who have missed those early-life opportunities. Further study is needed to understand other potential interventions that may mitigate the negative impact of ACEs in later-life.

## Author statement

**Marisa Nishio**: Conceptualization, Formal analysis, Writing- Original draft, Visualization.; **Michael Green**: Supervision, Writing- Reviewing and Editing.; **Naoki Kondo**: Data curation, Writing- Reviewing, Funding acquisition.

## Declaration of competing interest

MG was funded by the UK Medical Research Council (MC_UU_00022/2), and the 10.13039/100012095Scottish Government Chief Scientist Office (SPHSU17). This study used data from JAGES (the Japan Gerontological Evaluation Study), which was supported by Grant-in-Aid for Scientific Research (15H01972, 15H04781, 15H05059, 15K03417, 15K03982, 15K16181, 15K17232, 15K18174, 15K19241, 15K21266, 15KT0007, 15KT0097, 16H05556, 16K09122, 16K00913, 16K02025, 16K12964, 16K13443, 16K16295, 16K16595, 16K16633, 16K17256, 16K17281, 16K19247, 16K19267, 16K21461, 16K21465, 16KT0014, 17K04305, 17K04306, 25253052, 25713027, 26285138, 26460828, 26780328, 18H03018, 18H04071, 18H03047, 18H00953, 18H00955, 18KK0057, 19H03901, 19H03915, 19H03860, 19K04785, 19K10641, 19K11657, 19K19818, 19K19455, 19K24060, 19K20909, 20H00557) from JSPS (Japan Society for the Promotion of Science); Health Labour Sciences Research Grants (H26-Choju-Ippan-006, H27-Ninchisyou-Ippan-001, H28Choju-Ippan-002, H28Ninchisyou-Ippan-002, H30-Kenki-Ippan-006, H29-Chikyukibo-Ippan-001, H30-Jyunkankinado-Ippan-004, 19FA1012, 19FA2001, 21FA1012, 21K19635), Research project on health and welfare promotion for the elderly from the Ministry of Health, Labour and Welfare, Japan; the Research and Development Grants for Longevity Science from Japan Agency for Medical Research and development (AMED) (JP18dk0110027, JP18ls0110002, JP18le0110009, JP20dk0110034, JP21lk0310073, JP21dk0110037), the Research Funding for Longevity Sciences from National Center for Geriatrics and Gerontology (24-17, 24-23, 29-42, 30-30, 30-22, 20-19, 21-20); Open Innovation Platform with Enterprises, Research Institute and Academia (OPERA, JPMJOP1831) from the Japan Science and Technology (JST); a grant from the Japan Foundation For Aging And Health (J09KF00804), a grant from Innovative Research Program on Suicide Countermeasures (1-4), a grant from Sasakawa Sports Foundation, a grant from Japan Health Promotion & Fitness Foundation, a grant from Chiba Foundation for Health Promotion & Disease Prevention, the 8020 Research Grant for fiscal 2019 from the 8020 Promotion Foundation (adopted number: 19-2-06), and grants from Meiji Yasuda Life Foundation of Health and Welfare. The views and opinions expressed in this article are those of the authors and do not necessarily reflect the official policy or position of the respective funding organizations.
