# Supplementary material for: Roles of participation in social activities in the association between adverse childhood experiences and health among older Japanese adults
Source: SSM Popul Health. 2021 Dec 14;17:101000. doi: 10.1016/j.ssmph.2021.101000 (PMC8703060; doi:10.1016/j.ssmph.2021.101000)
Supplement: Multimedia component 1 [file mmc1.pdf]

SUPPLEMENTARY APPENDIX 1

Table A.1 Response rates to each question of interest among participants who were assigned to ACEs-related version questionnaire in wave 4, and joined wave 5 (n=12,271)

Surveyed year	*Question*	*Respondents*	*Response rate (%)*
2013-14	ACEs*	11,011	89.7%
2013-14	Social participation	11,106	90.5%
2013-14	age	12,271	100.0%
2013-14	Depression**	10,428	85.0%
2016-17	Depression**	10,057	82.0%
2013-14	Subjective health	11,953	97.4%
2016-17	Subjective health	11,968	97.5%
2016-17	Childhood SES	11,503	93.7%
2013-14	Marital status	12,026	98.0%
2013-14	Living status	11,723	95.5%
2013-14	Working status	11,308	92.2%
2013-15	Education history	12,126	98.8%
2013-14	Equivalised income	10,196	83.1%
2013-14	ADL	11,880	96.8%
2013-14	IADL	11,988	97.7%
2013-14	Current disease	11,548	94.1%

*Note: ACEs' question includes all 8 episides of childhood adversity

*Note: Depression questions include all 15 questions regarding depressive episodes

SUPPLEMENTARY APPENDIX 2

For each individual i, an exposure weight W_i^X was estimated by

$$W_i^X = \frac{P(X = x_i)}{P(X = x_i | C = c_i)}$$

where x_i denotes the probability of receiving an individuals' observed X (ACEs) and c_i denotes the individuals' baseline covariates C. The denominator of W_i^X is the predicted probability of a participant having (or not having) ACEs conditional on c_i. For those who had ACEs, the numerator of W_i^X is the overall proportion of those 'with' ACEs in the study population, whilst for those who did not have ACEs, the numerator of W_i^X is the overall proportion of those 'without' ACEs in the study population.

For each individual i, an intermediate exposure weight W_i^M is estimated by

$$W_i^M = \frac{P(M = m_i | X = x_i)}{P(M = m_i | X = x_i, C = c_i, C_{myi} = c_{myi})}$$

where m_i denotes the probability of taking the individuals' M (i.e. their observed level of social participation) and c_{myi} denotes individuals' intermediate covariates C_{my}. The denominator of W_i^M is the probability of having the value of m_i conditional on $X = x_i, C = c_i, C_{myi} = c_{myi}$ (i.e. their observed covariates). The numerator of W_i^M is the probability of having the value of m_i conditional only on $X = x_i$. These two weights, W_i^X and W_i^M, were multiplied to create each individuals' analysis weight:

$$W_i = W_i^X * W_i^M$$

The third model adopted marginal structural models (MSMs) (Tyler J. VanderWeele, 2015; VanderWeele, 2009) to estimate the controlled direct effects (CDEs) of ACEs on depressive symptoms and subjective health, adjusting for observed confounders. The estimate of interest, the CDE with a binary exposure X and where the value of the mediator (m) is set to 1 can then be expressed by

$$CDE(m = 1) \equiv E[Y(m = 1)|(X = 1)] - E[Y(m = 1)|(X = 0)]$$

This equation shows a comparison of two hypothetical worlds. The former $E[Y(m = 1)|(X = 1)]$ denotes the probability of receiving outcome Y where the exposure X is set to 1 and mediator M is set to 1. The latter $E[Y(m = 1)|(X = 0)]$ denotes the probability of receiving Y where X is set to 0 and M is set to 1. By keeping M fixed at 1, the direct effect of X, unmediated by M, can be obtained (Naimi et al., 2016; VanderWeele, 2011) .

SUPPLEMENTARY APPENDIX 3

Table A2 Association of each ACE with frequent social participation

reference=0 i.e. not experienced	*OR*	*95% CI*
Loss of parent(s)	1.02	[0.90 , 1.17]
Parents' divorce	0.68	[0.44 , 1.07]
Mother or father suffered a mental illness	0.35	[0.15 , 0.86]
Father was violent with mother	0.85	[0.64 , 1.15]
Was hit hard by mother/father causing an injury	0.98	[0.55 , 1.77]
Felt not loved by parents	0.87	[0.73 , 1.05]
Was told hurtful things or was insulted by parents	0.94	[0.72 , 1.21]
Had financial trouble	0.92	[0.82 , 1.03]

Note1: OR=Odds Ratio, CI=Confidence interval

Note2: age category and gender were adjusted

SUPPLEMENTARY APPENDIX 4

Participants were asked the following questions based on the GDS-15 and answered yes or no to each.

1) Are you satisfied with your current life?
2) Do you sometimes feel there is no point in living?
3) Do you think your energy for daily life or your interest in what's going on in the world has been decreasing?
4) Do you feel your life is empty?
5) Do you often feel bored?
6) Do you usually feel good?
7) Do you feel something bad is going to happen?
8) Do you think you are fortunate?
9) Do you often feel helpless?
10) Do you prefer staying at home over going out?
11) Do you think you are more forgetful than others?
12) Do you think life is wonderful?
13) Do you feel full of energy?
14) Do you think there is no hope in your life?
15) Do you think others are better off than you are?

REFERENCE

Naimi, A.I., Schnitzer, M.E., Moodie, E.E., & Bodnar, L.M. (2016). Mediation Analysis for Health Disparities Research. *Am J Epidemiol,* 184, 315-324.

Tyler J. VanderWeele, P.D. (2015). *Explanation in Causal Inference: Methods for Mediation and Interaction*.

VanderWeele, T.J. (2009). Marginal structural models for the estimation of direct and indirect effects. *Epidemiology,* 20, 18-26.

VanderWeele, T.J. (2011). Controlled direct and mediated effects: definition, identification and bounds. *Scand Stat Theory Appl,* 38, 551-563.
